# Autophagy deficiency in neurodevelopmental disorders

**DOI:** 10.1186/s13578-021-00726-x

**Published:** 2021-12-17

**Authors:** Zhiqiang Deng, Xiaoting Zhou, Jia-Hong Lu, Zhenyu Yue

**Affiliations:** 1 State Key Laboratory of Quality Research in Chinese Medicine, Institute of Chinese Medical Sciences, University of Macau, Macao SAR, 999078 China; 2 Department of Neurology, The Friedman Brain Institute, Icahn School of Medicine at Mount Sinai, New York, NY 10029 USA; 3 Department of Geriatrics, Xiangya Hospital, Central South University, Changsha, 410008 Hunan China

**Keywords:** Neurodevelopmental disorders, Autism, Neuronal autophagy, Neurogenesis, Synaptic development

## Abstract

Autophagy is a cell self-digestion pathway through lysosome and plays a critical role in maintaining cellular homeostasis and cytoprotection. Characterization of autophagy related genes in cell and animal models reveals diverse physiological functions of autophagy in various cell types and tissues. In central nervous system, by recycling injured organelles and misfolded protein complexes or aggregates, autophagy is integrated into synaptic functions of neurons and subjected to distinct regulation in presynaptic and postsynaptic neuronal compartments. A plethora of studies have shown the neuroprotective function of autophagy in major neurodegenerative diseases, such as Alzheimer’s disease (AD), Parkinson’s disease (PD), Huntington’s disease (HD) and amyotrophic lateral sclerosis (ALS). Recent human genetic and genomic evidence has demonstrated an emerging, significant role of autophagy in human brain development and prevention of spectrum of neurodevelopmental disorders. Here we will review the evidence demonstrating the causal link of autophagy deficiency to congenital brain diseases, the mechanism whereby autophagy functions in neurodevelopment, and therapeutic potential of autophagy.

## Introduction

Macroautophagy (hereafter referred to as autophagy) is a fundamental degradation pathway by which cellular components are degraded and recycled through the lysosome. The post-mitotic, long-living neurons of the brain rely on autophagy in removing dysfunctional protein aggregates and organelles to maintain neuronal homeostasis. In neurons, autophagy deficiency causes accumulation of ubiquitinated proteins, axon dystrophy, abnormal synaptic transmission, and subsequent neurodegeneration. Aberrant autophagic activity is associated with various human central nervous system (CNS) diseases including neurodegenerative and neurodevelopmental disorders [1, 2].

To date, a highly conserved set of autophagy related genes (*ATGs*) have been characterized along with their roles in the autophagy process [3]. Autophagy begins with the formation of UNC-51-like kinase (ULK1) complex which consists of ULK1, ATG13L, FIP200 and ATG101 [4]. In response to nutrition status of cells, the ULK1 complex can be phosphorylated by mammalian target of AMP-activated protein kinase (AMPK) or rapamycin complex 1 (mTORC1) to initiate or terminate autophagy, respectively [5]. In nutrient depletion condition, ULK1 is released from mTORC1 inhibition, and subsequently phosphorylates the components of Beclin1-VPS34 complex to regulate VPS34 kinase activity, which is required for phagophore nucleation [6, 7]. The expansion of phagophore depends on two ubiquitin-like conjugation systems which are mediated by ATG7 [8]. As an E1 like enzyme, ATG7 can conjugate ATG5 and ATG12 to form ATG5-12 complex which further binds to ATG16L1. With the assist of ATG16L1/ATG5-12 complex, ATG7 can also promote the conjugation of protein LC3-I to phosphoethanolamine to generate LC3-II, which can bind autophagosomal membranes and aid in cargo recruitment [9, 10]. Eventually, the autophagosome fuses with a lysosome to form an autolysosome, where the degradation of the cytoplasmic material and organelles happens in the presence of the lysosomal hydrolytic enzymes.

Growing works have demonstrates a link of aberrant signaling pathways including autophagy to neurodevelopmental disorders. Neurodevelopmental disorders are a multifaceted group of mental diseases, which are characterized by cognitive deficits and behavioral impairment. The most prevalent developmental disorders are autism spectrum disorders (ASDs) which are diagnosed primarily by deficits in social communication and interaction, as well as restricted and repetitive behaviors [11]. ASDs are often accompanied by other comorbidities including intellectual disability, motor deficits, and sensory processing abnormalities [11]. Numerous studies have revealed the etiology of ASDs which is attributed to genetic factors and non-genetic factors, such as environmental factors affecting the risk of ASDs development in a complementary manner [12, 13]. A subset of ASDs includes autism, tuberous sclerosis, fragile X syndrome, and others [14]. The causal link of autophagy impairment to neurodevelopmental disorders is highlighted by a recent report of deleterious, recessive variants of *ATG7* in human [15]. Here, we summarize the evidence linking autophagy dysregulation to neurodevelopmental disorders and review the concise roles of autophagy in neural development and synaptic function.

## Evidence for autophagy dysregulation in neurodevelopmental disorders

### Genetic mutations of autophagy-related genes in neurodevelopmental disorders

#### ASD

ASD is caused by the combination of genetic and environmental factors. The heritability of ASD is estimated at 60–90% by meta-analyses [16]. Human genetics studies from large cohorts of ASD patients and controls have identified many rare genetic variants including copy number variants (CNVs) and single nucleotide variants (SNVs) associated with ASD [17, 18]. By applying eXome Hidden Markov Model (XHMM) to an ancestry-matched sample of ASD cases and controls, *Buxbaum* and his colleagues previously identified an increase in small CNV in ASD cases [19]. By performing enrichment and pathway analyses of the genes disrupted by deletions in ASD cases, they observed significant enrichment of five autophagy related genes including *GABARAPL2*, *GABARAPL1*, *MAP1LC3A*, *GABARAP*, and *MAP1LC3B*, which are mammalian orthologs to yeast autophagy gene *Atg8* [19]. The study implicates dysregulation of autophagy in ASD. In support of the notion, a recent study has reported that aggregation of GABARAPs is increased in the postmortem brain of ASD patients and that depletion of autophagy in forebrain GABAergic interneurons in adolescent mice causes the formation of high-molecular weight species of GABARAPs [20]. These mice with autophagy deficiency display an overlapping set of ASD-like social behavioral impairment [20].

#### Genetic leukoencephalopathies

Genetic leukoencephalopathies are a set of heterogeneous disorders characterized by brain white matter defects in central nervous system (CNS), motor impairment, ataxia, and impaired cognitive development [21]. VPS11 protein, encoded by *VPS11* gene, is a core component of CORVET (class C core vacuole/endosome tethering) protein complexes which is involved in membrane trafficking and lysosome-endosome fusion. By using whole exome sequencing, our recent study reported a homozygosity for a missense variant of *VPS11* (C846G) in five individuals with leukoencephalopathy syndrome [14]. The study further indicates that C846G mutation in *VPS11* displays a loss of function in autophagy pathway in human cells and that zebrafish carrying a *VPS11* mutation shows a significant reduction in CNS myelination [14]. The study reveals a defect in *VPS11*-mediated autophagy-lysosome trafficking pathway as an underlying mechanism for some forms of leukoencephalopathy.

#### Childhood ataxia

Childhood ataxia is a rare disease leading to difficulties in coordination and movements, as well as cognitive problems and developmental delay in children [22]. A recent study has identified a homozygous missense mutation in *ATG5*, a core gene in autophagy, in two siblings with congenital ataxia [22]. The cells derived from the subjects display defects in autophagy activity [22]. The yeast harboring homozygous mutation of *ATG5* show lower levels of autophagy compared to normal cells [22]. Further experiments demonstrates that introducing wild-type human *ATG5*, but not mutated form, into fruit flies lacking fly *ATG5* can restore normal movement [22]. The findings implicate that the mutation in *ATG5* is responsible for the symptoms of childhood ataxia.

#### Primary microcephaly

Primary microcephaly is a congenital neurodevelopmental disorder characterized by reduced head circumference and brain volume [23]. By using the whole exome sequencing analysis, a recent study has identified a dominant mutation in *ALFY*, encoding an autophagy scaffold protein, as a causative mutation to primary microcephaly [23]. The results show that transgenic flies with overexpression of the mutant human *ALFY* recapitulate the phenotype of microcephaly in human patients [23]. Further experiments demonstrate that wild-type *ALFY*, but not mutant human *ALFY*, controls the removal of DVL3 aggregates to regulate Wnt signaling [23]. Recently, *Mason *et al. have reported that eliminate the expression of *ALFY* in mice brain causes developmental axonal connectivity and impairs the formation of the major forebrain commissures [24]. Collectively, these findings suggest ALFY-mediated autophagy plays a critical role in the development of human brain and microcephaly.

#### Complex developmental disorders

*WIPI2*, the mammalian homologue of the yeast *Atg18*, is a key regulator of autophagy. WIPI2 interacts with ATG16L1 and recruits the ATG12-ATG5-ATG16L1 complex to the phagophore and therefore promotes LC3 lipidation and subsequent autophagosome formation [25]. By performing whole exome sequencing on affected individuals with a complex developmental disorders including mental retardation, speech and language impairment, as well as other neurological and psychiatric abnormalities, a recent study identified a novel nonsynonymous homozygous mutation (V249M) in *WIPI2* gene [26]. The same study reported that V231M mutation on *WIPI2b* (corresponding to V249M in *WIPI2a*) significantly reduced its interaction with ATG16L1 and ATG5-12 complex [26]. Compared to the controls, the fibroblasts derived from the patients carrying the V249M mutation show reduced LC3 lipidation, which is correlated to the reduced WIPI2 puncta, and subsequent reduced level of autophagy flux [26]. The results imply that the impairment of autophasosome formation may cause the neurodevelopmental disorders. In line with this notion, by performing whole exome sequencing on a family in which one of four children displays severe cortical atrophy, intellectual impairment, ataxia, and other neurological symptoms, *Keays *et al. identified a single homozygous coding mutation (L1224R) in *VPS15* in an affected case [27]. VPS15 is a key component in VPS15-VPS34-Beclin1 complex which plays a critical role in autophagosome formation. The experiments performed by *Keays *et al. demonstrate that compared to controls, the dermal fibroblasts derived from affected individual show reduced protein levels of VPS15, VPS34 and Beclin1, decreased LysoTracker staining, and increased protein level of p62, an autophagy cargo receptor [27]. Further study indicates that ectopic expression of wild-type *VPS15* in L1224R patient cells increases the protein levels of VPS15, stabilizes VPS34 and Beclin1, and decreases the protein level of p62 [27]. These findings indicate that L1224R mutation in *VPS15* is associated with human neurodevelopmental disorders through compromising the function of VPS15-VPS34-Beclin1 complex in autophagy.

ATG7 is an essential effector enzyme for canonical autophagy. Most recently, by performing genetic and clinical analysis, *Taylor *et al. identified recessive and loss-of-function mutations in both *ATG7* alleles in 12 individuals from five unrelated families, which exhibit complex neurodevelopmental disorders including ataxia and developmental delay [15]. Experiments conducted in the fibroblasts and skeletal muscles derived from the patients indicate that the expression of *ATG7* is diminished or absent in patients derived cells, resulting in impaired LC3 lipidation and autophagy flux [15]. The functional complementation experiments in mice and yeast confirmed the functional deficiencies induced by the missense variants in *ATG7* [15]. Taken together, the study reveals the critical role of basal autophagy in human neural development and integrity.

### Autophagy dysregulation in neurodevelopmental disorders

Growing evidence indicates the dysregulation of mTOR in ASD [28–30]. mTOR is a central regulator of diverse cellular processes including autophagy. mTOR is negatively regulated by tuberous sclerosis complex 1/2 (TSC1/2) [16, 31, 32]. Previous study reported that the *TSC2* ± mice display constitutive hyperactivity of mTOR, blockade of autophagy, and consequent spine pruning defects [33]. Moreover, an mTOR inhibitor rapamycin can correct the spine pruning defects and ASD-relevant behaviors in *TSC2* ± mice, but not in *TSC2*±:*ATG7 *^*cKO*^ mice [33]. A most recent study has reported the similar results in parvalbumin (PV) cell-restricted *TSC1* conditional haploinsufficient and knockout mice, which show transient autophagy dysfunctions, a loss of perisomatic innervation and social behavior deficits [34]. Moreover, treatment with rapamycin in a sensitive period rescues PV cell connectivity and social behavior in *TSC1* conditional haploinsufficient mice [34]. Apart from TSC1/2 models of ASD, recent studies have reported the impaired expression of autophagy related protein Beclin1 in animal models of ASD including *Cc2d1a* ± and *ADNP* ± mice [35, 36]. These studies indicate that dysregulation of autophagy may contribute to neuronal pathology and aberrant social behaviors in ASD.

Fragile X syndrome (FXS), a leading genetic cause of autism, is a heritable form of intellectual disabilities including autistic behaviors, attentional deficits, emotional lability, impaired cognition and other neurological disabilities [37–39]. Fragile X mental retardation (*Fmr1*) is a causative gene to FXS [40]. Fragile X mental retardation protein (FMRP), encoded by *Fmr1* gene, is an RNA-binding protein that tightly regulates the function of multiple neuronal mRNA critical to neuronal development and synaptic plasticity [41, 42]. *Fmr1*-KO mice is a well-characterized model of FXS [40]. Previous studies have reported the dysregulation of mTOR signaling in FXS mice and in humans with FXS [43, 44]. A recent study demonstrates that the biochemical markers of autophagy such as LC3II puncta, the active form of p-ULK1 and p-Beclin1, and consequent autophagy flux are significantly reduced, while p62 is accumulated, in the hippocampal neurons of *Fmr1*-KO mice, perhaps as a result of deregulated mTOR signaling [45]. Mechanistic investigations indicate that the mTORC1 activity is enhanced and Raptor, a defining component of mTORC1, translocates to lysosome [45]. And specific knockdown of Raptor in the hippocampal neurons activates autophagy and rescues the impaired synaptic plasticity and cognition in *Fmr1*-KO mice [45]. The findings indicate that the mTOR-dependent autophagy is impaired in FXS and activation of autophagy through mTOR inhibition prevents the neuronal deficits in FXS.

Recent studies have reported dysregulation of mTOR-dependent autophagy in other neurodevelopmental disorders including Schaaf-Yang syndrome (SHFYNG) and Koolen-de Vries syndrome (KdVS) [46, 47]. SHFYNG is a neurodevelopmental disorder caused by *MAGEL2* mutations and the patients with SHFYNG show feeding difficulties, intellectual disability and cognitive impairment, and increased prevalence of ASD [48–50]. *Schaaf *et al. reported that the mTOR activity is increased, accompanied by decreased autophagy flux in *MAGEL2* null mice and fibroblasts derived from SHFYNG patients [46]. The induced pluripotent stem cell (iPSC)-derived neurons from SHFYNG patients show impaired dendrite formation which can be rescued by treatment with rapamycin [46]. KdVS is neurodevelopmental disorder caused by mutations with loss-of-function in *KANSL1* gene and patients with KdVS manifest epilepsy, congenital malformations and developmental delay [51–53]. Most recently, *Kasri *et al. have reported that iPSC-derived neurons from KdVS patients display accumulated autophagosome at excitatory synapses, resulting in reduced synaptic density and impaired neuronal network activity [47]. Mechanistically, they found that in these iPSC-derived neurons, the mTOR activity is enhanced and the lysosome function is decreased, thus preventing the clearance of autophagosome [47]. Taken together, these findings indicate that the mTOR-dependent autophagy is disrupted in these neurodevelopmental disorders.

## Autophagy controls neurogenesis

The evidence that loss-of-function mutations in essential autophagy genes causes the neurodevelopmental disorders demonstrate a crucial role of autophagy in neurodevelopment. What is the mechanism underlying the function of autophagy in controlling neurodevelopment? Autophagy is constitutively active in the development of CNS [54]. Through digesting the toxic proteins or aggregates and damaged organelles, autophagy critically regulates neuronal plasticity during neuronal development. Given the growing interest in the role of autophagy in neural proliferation and in maintenance of neuronal stem cells (NSC), here we review the evidence linking autophagy to neurogenesis.

By using knockout strategies, previous studies have investigated the role of autophagy in embryonic neurogenesis [55]. *Jiao *et al. have shown a crucial role of autophagy in cortical neurogenesis during early brain development [56]. They found that the *ATG5* expression increased during cortical development and differentiation [56]. Suppression of *ATG5* by using electroporation of short hairpin shRNAs causes reduced neural progenitor cells (NPCs) differentiation, and consequent impaired morphology of cortical neurons [56]. Mechanistic investigations indicate that ATG5-mediated autophagy regulates β-Catenin signaling pathway, which is critical for NPCs proliferation and differentiation in neurodevelopment. They showed that autophagy cooperates with β-Catenin to modulate the proliferation and differentiation of cortical NPCs in embryonic neurogenesis during brain development [56]. Furthermore, another study reported that depletion of *ATG5* represses astrocyte differentiation in vitro and in the developing mouse cortex, whereas overexpression of *ATG5* enhances astrocyte differentiation [57]. Additional evidence indicated that through promoting the degradation of SOCS2, ATG5-mediated autophagy activates the JAK2-STAT3 signaling, which regulates the differentiation of astrocyte, while the impaired astrocyte differentiation caused by *ATG5* deficiency can be rescued by SOCS2 knockdown [57]. These studies indicate that ATG5-mediated autophagy regulates both neurogenesis and gliogenesis during early brain development.

*FIP200* (also known as *Rb1cc1*) is an essential gene for autophagy induction. Guan’s group previously reported that depletion of FIP200 causes a progressive loss of NSCs and impairs neuronal differentiation in postnatal brain of mice, which can be rescued by treatment with the antioxidant N-acetylcysteine [58]. Recently, the same group found evidence that microglial dysregulation contributes to the impairment of neurogenesis in *FIP200*-null NSCs from *FIP200*;*p53*hGFAP 2cKO mice [59]. Mechanistically, the study reveals that ablation of FIP200 leads to increased infiltration of microglia into the subventricular zone and subsequent microglia activation in *FIP200*; *p53*hGFAP 2cKO mice [59]. Inhibition of microglia infiltration and activation can rescue the defective neurogenesis in these 2cKO mice [59]. These findings demonstrate that FIP200-mediated autophagy plays a critical role in regulating neurogenesis in postnatal NSCs through the control of microglia migration and activation.

In addition to embryonic neurogenesis, adult neurogenesis can also be regulated by autophagy [60]. The Notch signaling pathway has an important role in adult neurogenesis [61]. Previous studies have shown that activation of Notch signaling inhibits proliferation and differentiation of NSCs in adult brain [62, 63]. *Rubinsztein*’s group has recently reported that Notch1, a plasma membrane-resident receptor in Notch signaling, is degraded by autophagy through ATG16L1, a crucial autophagy protein [64]. They demonstrated that ATG16L1 protein level was reduced, whereas the levels of Notch signaling proteins Notch1, NICD and Hes1 are significantly increased in the brain of *ATG16L1*-hypomorph mice [64]. They further showed decreased differentiation of NSCs and smaller cortical plate in *ATG16L1*-hypomorph compared with the control mice [64]. Using BrdU labeling, they showed that the number of BrdU-positive cells is significantly reduced in *ATG16L1*-hypomorph mice compared with the controls at 9–11 months old [64]. The results indicate that ATG16L1-mediated autophagy controls both embryonic and adult neurogenesis through regulating the degradation of proteins in Notch signaling pathway.

The Forkhead Box O (FOXO) proteins are a class of conserved transcription factors that control gene expression programs involved in multiple cell signaling pathways [65]. Previous studies have reported the roles of FOXO proteins in the regulation of adult NSCs homeostasis and autophagy induction [66–68]. A recent study has demonstrated that FOXO3 directly regulates autophagy pathway to maintain the proteostasis in adults NSCs [69]. *Wurst *et al. recently reported that conditional knockout of FOXO1/3/4 in adults NSCs by using GLAST::CreERT2 impairs autophagy flux both in in vitro and in vivo [70]. FOXO1/3/4 deficiency leads to altered dendrite and spine development of adult-generated neurons and impaired long-term survival eventually [70]. Further evidence indicates that autophagy inducer rapamycin not only rescues the impaired autophagy flux in NSCs, but also reverses the impaired phenotypes of dendrite and spine of adult-generated neurons with FOXO1/3/4 deficiency [70]. These findings indicate that FOXO proteins regulate neuronal morphogenesis via the maintenance of autophagy flux during adult neurogenesis.

## Autophagy regulates presynaptic and postsynaptic development and synaptic activity

Neurons are postmitotic cells which are maintained for the lifetime of the organism. However, the synapses of neurons are highly dynamic, especially during early lifetime as the growing rate of synapses experiences an initial increase and subsequent decrease before an eventual stabilization of synapse development. During the synapse development the properties of synapses can be changed and reshaped, while new neural circuits are developed under circumstances such as learning and stress. Available evidence showed that autophagy plays a role in synapse formation and pruning, a process facilitating the removal of exuberant neuronal connections [71]. In addition, autophagy may also regulate synaptic activity, the function of which depends on the synapse transmission and plasticity.

### Autophagy regulates presynaptic and postsynaptic development

During early development, autophagy is required for axon pathfinding and synaptic vesicle clustering formation during early synaptogenesis [72–74]. Previous studies showed that loss of autophagic scaffolding protein ALFY leads to a failure in axon guidance and outgrowth in the development of mouse brain [75]. Neural-specific depletion of ATG9 results in abnormal development of axon tracts in mouse brain regions including the corpus callosum and anterior commissure [73]. In *C.* *elegans* interneuron, autophagy controls presynaptic assembly and axon outgrowth dynamics, which is spatially regulated through the coordination of ATG9 and synaptic vesicle kinesin, KIF1A/UNC-104 [76]. Consistent with this notion, a previous study conducted in Drosophila indicated that *Atg1* (an ortholog of ULK1 in S. cerevisiae) mutant causes reduced total neuromuscular junction (NMJ) area and decreased number of synapses [77]. However, overexpression of wildtype *Atg1* increases NMJ synaptic bouton number in an autophagy-dependent manner [77].

In the postsynaptic site, autophagy was shown to be involved in synaptic pruning. Deficiencies in autophagy result in an overabundance of dendritic spines, ultimately manifesting as autism-like phenotypes [33]. For example, the primary hippocampal neuron cultures with reduced *Atg7* expression showed increased PSD95 density, a marker for postsynaptic abundance [33]. The similar results were shown in mouse models with *Atg7* knockdown [33]. Another study showed that NMJs from autophagy deficient neurons exhibited postsynaptic folds without presynaptic axon terminal opposed to them, suggesting an abnormal postsynaptic pruning [78]. These results indicate that basal autophagy may play an important role in specific postsynaptic receptor degradation and postnatal spine pruning.

### Autophagy regulates synaptic activity

Multiple lines of evidence show that autophagy modulates neurotransmitter release and neural plasticity. *Marijn Kuijpers *et al. recently found that the neurotransmission and calcium sensitivity is significantly increased in primary hippocampus excitatory neurons in *Atg5* knock-out mouse due to the deregulation of ER turnover [79]. Moreover, a previous study found that depletion of autophagy by using a dopaminergic neuron-specific *Atg7* knock-out mouse model significantly affects dopamine release and reuptake [80]. The study further showed that striatal slices from *Atg7* DAT::Cre mice had increased evoked dopamine release in cyclic voltammetry experiments and enhanced presynaptic recovery following paired-pulse stimulation. Moreover, rapamycin treatment reduced stimulus-evoked dopamine release in slices from *Atg7* DAT::Cre mice [80]. These studies provide evidence for the role of presynaptic autophagy in the regulation of neurotransmission.

**Fig. 1:**
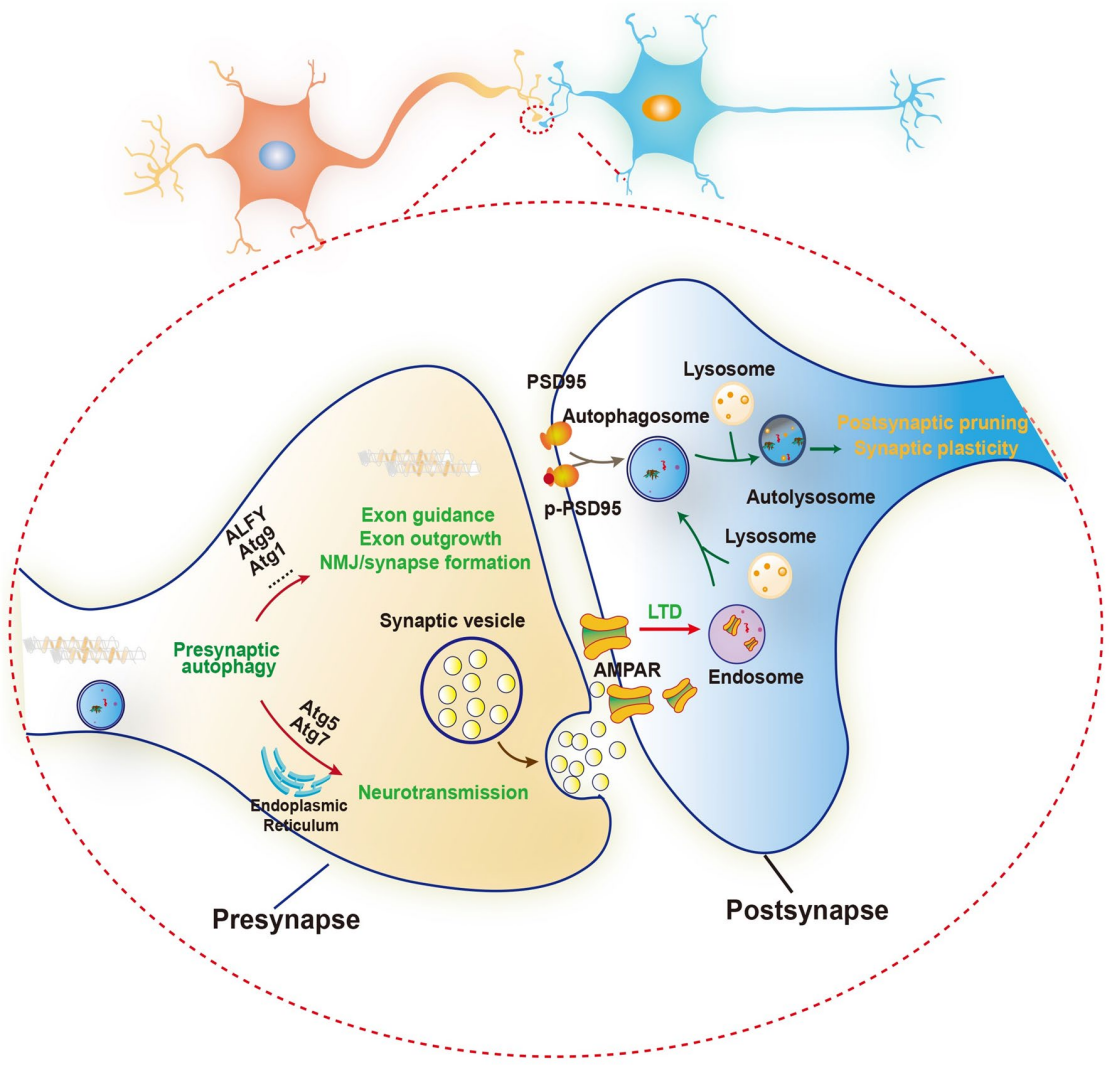
Autophagy regulates synaptic development and activity. In the presynaptic site, autophagy genes, such as ALFY, Atg9 and Atg1, regulate exon guidance and outgrowth, neuromuscular junction (NMJ) and synapse formation. Atg5 and Atg7 regulate neurotransmission. Atg5 mediated autophagy regulates neurotransmission through regulation of ER turnover. In the postsynaptic site, autophagy mediates synaptic pruning through the degradation of PSD95 and phosphorylated PSD95. Besides, autophagy regulates NMDAR-LTD by the removal of AMPA receptors from postsynaptic membranes to late endosomes for degradation to control synaptic plasticity

A recent study showed that autophagy regulates development-related synaptic plasticity and memory [81]. NMDA receptor-dependent long-term depression (NMDAR-LTD) is a long-lasting form of synaptic plasticity [82]. The induction of NMDAR-LTD is mediated by the removal of AMPA receptors from postsynaptic membranes to late endosomes for degradation [83]. It is induced in early stage of neuronal development while greatly reduced in adulthood during CNS development. Down-regulation of NMDAR-LTD in adults is physiologically significant for memory formation. *Shen *et al. revealed that autophagic flux in CA1 neurons was transiently decreased during the induction phase of NMDAR-LTD. Autophagy inhibition caused a reduction of endocytic recycling and is required for AMPA receptor internalization and synaptic depression in mouse CA1 neurons. In adulthood, autophagy is up-regulated to decrease the inducibility of LTD, thereby preventing the adverse effect of excessive LTD on memory consolidation [81]. Additionally, *Compans *et al. found that autophagy is required for the degradation of T19-phosphorylated form of PSD95 in NMDAR-LTD induction, which triggers a depletion of PSD95 from synapses and eventually increases short-term plasticity to improve neuronal responsiveness of depressed synapses [84]. A most recent study has shown that constitutive induction of mTOR-dependent autophagy rescues the prevention of NMDAR-LTD induced by disrupting synergistic action of CREB and CRTC1, two essential transcriptional factors for late-phase long-term synaptic potentiation [85]. These findings reveal the previously unrecognized functions of autophagy in the regulation of synaptic plasticity and memory (Fig. 1).

## Conclusive remarks

Growing evidence has highlighted the important role of autophagy in regulating neurodevelopment and synaptic plasticity. Alteration of autophagy may lead to the abnormal neurodevelopment and malfunction of synapses in the brain. Recent human genetic and clinical studies have identified the link of congenital mutations in key autophagy related genes to neurodevelopmental disorders (Table 1). However, the causality of the autophagy deficiency to the disease awaits further clarification due to non-autophagy functions associated with many autophagy related genes. It is also extremely challenging to address the specific role of autophagy because of the difficulty of monitoring autophagy flux directly in human brain. While rodent models are valuable tools to dissect the role for autophagy in neurodevelopment and differentiation (e.g., engineering autophagy gene deletion in CNS), the possibly differential functions of autophagy homologous genes between rodent and human were noticed and may complicate the interpretation of the results [86].

**Table 1:** Genetic mutations of autophagy-related genes in neurodevelopmental disorders

Gene symbol	Protein	Autophagy involvement	Genetic variant	The effect of genetic variant	Neurodevelopmental disorder
*MAP1LC3A*, *MAP1LC3B*, *GABARAP*, *GABARAPL1*, *GABARAPL2*	MAP1LC3A, MAP1LC3B, GABARAP, GABARAPL1, GABARAPL2	Cargo recruitment	None	Loss of function in autophagy pathway	ASD [19]
*VPS11*	VPS11	Membrane trafficking and lysosome-endosome fusion	C846G	Loss of function in autophagy pathway	Genetic leukoencephalopathy [14]
*ATG5*	ATG5	Elongation	E122D	Lower levels of autophagy	Childhood ataxia [22]
*ALFY*	ALFY	Autophagy adaptor	R2637W	Loss of function in removal of aggregates	Primary microcephaly [23]
*WIPI2*	WIPI2	Nucleation	V249M	Reduced LC3 lipidation and autophagy level	Complex developmental disorder [23]
*VPS15*	VPS15	Nucleation	L1224R	Increased protein level of p62	Complex developmental disorder [27]
*ATG7*	ATG7	Elongation	R659*	Impairment in LC3 lipidation and autophagy flux	Complex developmental disorder [15]
			R576H		
			H624Y		
			P234T		
			V588M		
			Q261R		
			G511D		
			L512P		

A most recent study has identified recessive and loss-of-function mutations in both *ATG7* alleles in patients with neurodevelopmental disorders in five unrelated families [15]. Despite the complete absence of ATG7 protein, the patients carrying the missense variants of *ATG7* had approached population life expectancy [15, 87]. In contrast, mice lacking *Atg7* gene die early postnatally. The data suggests that humans are much more tolerant to the loss of *ATG7* or ATG7-mediated autophagy. Alternative explanation is that cellular functions that can compensate for the loss of ATG7 function in survival are more robust in humans than rodents. To dissect directly ATG7-mediated autophagy in neurodevelopment in humans, future experiment should use human neurons carrying corresponding mutants. The human neurons derived from induced pluripotent stem cells (iPSCs) would provide an important model to investigate the mechanism whereby autophagy deficiency leads to neurodevelopmental disease, and to test therapeutic strategy by restoring autophagy function.

## Data Availability

Not applicable.
